# The role of *Enterococcus* spp. and multidrug-resistant bacteria causing pyogenic liver abscesses

**DOI:** 10.1186/s12879-017-2543-1

**Published:** 2017-06-26

**Authors:** Marcus M. Mücke, Johanna Kessel, Victoria T. Mücke, Katharina Schwarzkopf, Michael Hogardt, Christoph Stephan, Stefan Zeuzem, Volkhard A. J. Kempf, Christian M. Lange

**Affiliations:** 1 Department of Internal Medicine 1, University Hospital Frankfurt, Frankfurt am Main, Germany; 2 University Center for Infectious Diseases, University Hospital Frankfurt, Frankfurt am Main, Germany; 3 Department of Internal Medicine 2, University Hospital Frankfurt, Frankfurt am Main, Germany; 4 Institute of Medical Microbiology and Infection Control, University Hospital Frankfurt, Frankfurt am Main, Germany

**Keywords:** Pyogenic liver abscess, Bacterial pathogens, Susceptibility profiles, Multidrug-resistant organisms, Vancomycin-resistant enterococci

## Abstract

**Background:**

Pyogenic liver abscesses (PLA) remain a significant clinical problem. Unfortunately, little is known about current bacterial susceptibility profiles and the incidence of multidrug resistant organisms (MDROs) causing PLA in Western countries. Yet, this crucial information is pivotal to guide empirical antibiotic therapy. Aim of this study was to provide detailed characteristics of PLA with a special focus on underlying bacterial pathogens and their susceptibility to antibiotics.

**Methods:**

A retrospective study of patients diagnosed with PLA from 2009 to 2015 in a large tertiary reference center in Germany was performed in order to characterize PLA and antimicrobial susceptibility profiles of causative bacterial species.

**Results:**

Overall, 86 patients were included. The most common causes of PLA were bile duct stenosis/obstruction (31.4%) and leakage of biliary anastomosis (15.1%). Frequent predisposing diseases were malignancies (34.9%), diabetes (24.4%) and the presence of liver cirrhosis (16.3%). Of note, *Enterococcus* spp. were the most frequently cultured bacterial isolates (28.9%), and in 1/3 of cases vancomycin resistance was observed. In addition, a relevant frequency of gram-negative MDROs was identified. In particular, an alarming 10% and 20% of gram-negative bacteria were resistant to carbapenems and tigecycline, respectively. Of note, MDRO status did not predict ICU stay or survival in multivariate regression analysis. The mortality rate in our series was 16.3%.

**Conclusion:**

Our study demonstrates an as yet underreported role of *Enterococcus* spp., often associated with vancomycin resistance, as well as of gram-negative MDROs causing PLA.

**Electronic supplementary material:**

The online version of this article (doi:10.1186/s12879-017-2543-1) contains supplementary material, which is available to authorized users.

## Background

With an annual incidence of 1.1 to 2.3 per 100,000 and mortality rates of up to 12% in developed countries, pyogenic liver abscesses (PLAs) remain a significant clinical problem in the Western World [1–4]. Even higher incidence rates have been reported in Asian countries, e.g. in Taiwan (17.06 cases per 100,000) [5]. Due to various severe predisposing diseases (e.g. biliary strictures or cancer), the frequent need of external and internal drainage, and a plethora of potential causative microorganisms, medical management of PLA can be highly complex.

In general, multidrug-resistant organisms (MDROs), including vancomycin-resistant enterococci (VRE), methicillin-resistant *Staphylococcus aureus* (MRSA) or multidrug-resistant gram-negative bacteria (MRGN), are increasingly being observed worldwide [6, 7]. Growing resistance in particular among certain gram-positive and gram-negative pathogens – so-called “ESKAPE” pathogens (*Enterococcus faecium*, *Staphylococcus aureus, Klebsiella pneumoniae, Acinetobacter baumannii, Pseudomonas aeruginosa*, and *Enterobacter* species [8]) – causing infections in hospitals and in the community are worrysome. Of special concern are reports portraying a growing number of organisms resistant to all available antibiotics, including polymyxin [7, 9–11]. Recently, several case reports of PLA caused by MDROs have been published [12, 13]. In addition, Lo et al. noted in a series of Asian patients with PLAs an increase of MDR isolates (*Klebsiella pneumoniae*) from 1.6% to 14.3% within 10 years in Singapore [14]. In contrast, current data from Western countries are largely lacking.

Early retrospective studies have revealed remarkable differences between PLA characteristics in Asian and Western countries [1–4, 14–16]. For example, *Klebsiella pneumoniae* has been identified as the predominant cause of PLA in Asia [5, 17–19], whereas other *Enterobacteriaceae* such as *E. coli*, as well as *Staphylococcus* spp.*, Streptococcus* spp., *Enterococcus* spp., or anaerobes were predominantly isolated in the Western World [1–4]. Unfortunately, little is known about prevailing susceptibility profiles and the incidence of MDROs causing PLA in the Western countries. However, this crucial information is pivotal to guide antibiotic therapy, one of the fundaments of PLA treatment.

In this retrospective study we therefore aimed to further describe PLA characteristics including a detailed analysis of current bacterial and fungal isolates causing PLA in a large tertiary reference center in Germany. Our study reveals a so far underestimated role of *Enterococcus* spp. and MDRO in secondary PLA and thereby helps to guide empirical antibiotic therapy of PLA.

## Methods

### Study population

All adult patients admitted to the University Hospital Frankfurt, Germany, between January 2009 and December 2015 with the discharge diagnosis of PLA were eligible for inclusion. For identification of possible patients, the patient chart database of the University Hospital Frankfurt was systematically searched for code K75.0 or K83.0 of the International Classification of Diseases, Tenth Revision, German Modification. Cases were included if (1) one or more discrete hepatic abscess cavities were confirmed by at least one imaging modality – ultrasound (US), computed tomography (CT) or magnetic resonance imaging (MRI) – as well as (2) by either positive culture results retrieved from the abscess or resolution of symptoms after antibiotic therapy. Patients were excluded if they were younger than 18 years old, if parasitic/amoebic abscesses were diagnosed or if available data were incomplete. The local ethics committee approved this study.

### Clinical data collection, definitions

Charts were systematically reviewed and information obtained was gathered in a data collection form. Information recorded included sex, age, date of admission/discharge, underlying medical condition, initial symptoms and the intake of immunosuppressant agents, antibiotics, and proton-pump inhibitors. Additionally, initial laboratory values were documented. Laboratory results were considered to be the first values obtained upon hospital admission due to PLA or within 24 h upon presentation of PLA when PLA was not the initial cause of hospitalization. Imaging reports (CT, MRI, US) were analyzed and number and size of PLAs were documented.

For conventional microbiological culture procedures, aerobic and anaerobic conditions including the use of thioglycolate enrichment medium were applied. Species identification of recovered microorganisms was performed by matrix-assisted laser desorption/ionization time-of-flight (MALDI–TOF) mass spectrometry (VITEK MS, bioMérieux, Nürtingen, Germany) and VITEK2 (bioMérieux, Nürtingen, Germany). Antibiotic susceptibility testing (AST) was done by VITEK2 (bioMérieux, Nürtingen, Germany) according to Clinical and Laboratory Standards Institute (CLSI) guidelines and/or antibiotic gradient tests (Etest), where necessary. All laboratory tests were performed under strict quality-controlled criteria (laboratory accreditation according to ISO 15189:2007 standards; certificate number D–ML–13102–01–00, valid through January 25th, 2021).

In the vast majority of cases, positive microbiological results of abscess cavity cultures were obtained. A bacterial isolate was considered to be an MDRO if it belonged to either category VRE, MRSA or MRGN. MRGN status was defined according to the German KRINKO guideline [20]. When microbiological results were available the initial empiric antibiotic treatment was assessed and considered to be adequate if the retrieved isolates were testes to be susceptible. Additionally, mycotic coinfections were documented.

The responsible physician defined the assumed cause of PLA. The therapeutic modality was classified as either surgery, percutaneous drainage (either CT- or US-guided), biliary drainage by endoscopic retrograde cholangiography (ERCP) or percutaneous transhepatic cholangiography and drainage (PTCD), or solitary medically managed. Complications and outcome, e.g. treatment on an intensive care unit (ICU), recurrence of abscesses, mortality and duration of hospitalization, were also recorded.

Finally, local hospital surveillance data was analyzed to compare rates of *Enterococcus* spp. and VRE causing PLA with the overall documented infection rates of *Enterococcus* spp. and VRE in our gastroenterology/hepatology wards between 2010 and 2015. In brief, microbiological data were extracted from the hygiene software HyBASE 6.1 (epiNET, Germany) and cross-checked by the laboratory Information system Swisslab 7.1.3 (Roche Diagnostics IT solutions, Germany). For the calculation of the relative VRE rate, *E. faecium* and/or *E. faecium*-VRE positive cultures of all microbiological specimens submitted during 2010–2015 to the laboratory were counted. Patients with an infection where both VRE and Enterococcus were isolated were counted as patients with a VRE infection only to avoid duplicates.

### Statistical analysis

For statistical analysis BiAS, Version 11.03, was applied.

Group differences were calculated using the nonparametric Mann-Whitney U test (continuous variables) or Fischer’s exact (categorical variables), as appropriate. After bivariate/univariate analysis, multivariate analysis was performed by using backward selection and a *P* value ≥0.10 for removal from the model. Only patients with complete data for the remaining covariates were included in multivariate analysis. Sex and age were forced into the model. Odds ratios (OR) and respective 95% confidence intervals (CI) were calculated for each variable. All statistical tests were two-sided and *P* values <0.05 were considered to be significant.

## Results

### Patient characteristics

**Fig. 1:**
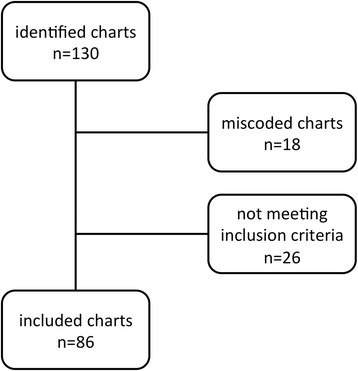
Study overview

From 130 identified charts, 86 patients (55 men and 31 women, median age 62 years [IQR 51–72 years]) matching the described criteria were included in this study (Fig. 1). Detailed clinical characteristics and laboratory results as well as symptoms of patients, underlying diseases, direct cause and abscess’ characteristics are displayed in Tables 1 and 2, respectively.

**Table 1:** Age, duration of hospitalization and laboratory abnormalities among patients with pyogenic liver abscess

Characteristics	Median (IQR)	Cases with abnormal values, %
Age (years, *n* = 86)	62 (51–72)	...
Duration of hospitalization (days, *n* = 86)	20 (11,3–36,5)	...
CRP (mg/dl, *n* = 86)	10,9 (7,1–17,4)	98,8
WBC (/nl, *n* = 86)	11,1 (7,5–16,3)	77,9
AST (U/l, *n* = 85)	42 (27–102)	61,6
ALT (U/l, *n* = 86)	36 (19–73)	51,2
Bilirubin (mg/dl, *n* = 85)	0,9 (0,6–3,6)	36,0
Albumin (g/dl, *n* = 81)	2,8 (2,3–3,1)	84,9
γGT (U/l, *n* = 86)	199 (93–420)	91,9
AP (U/l, *n* = 86)	219 (135–386)	75,6
Creatinine (mg/dl, *n* = 86)	0,8 (0,6–1,0)	19,8
INR (*n* = 86)	1,3 (1,1–1,4)	48,8

**Table 2:** Clinical characteristics of patients, underlying diseases and abscess’ characteristics

Variable	No. of patients (%)
Comorbidities^a^	
Malignancy	32 (34.9)
Diabetes mellitus	21 (24.4)
Liver cirrhosis	14 (16,3)
Liver transplantation	10 (11.6)
Direct cause of abscess	
Bile duct stenosis/obstruction	27 (31.4)
Anastomosis leakage	13 (15.1)
Biliary infection	13 (15.1)
Superinfected liver metastasis	8 (9.3)
Ischemic	5 (5.8)
Intra-abdominal infection	2 (2.3)
Non-intestinal sepsis	4 (4.7)
Cryptogenic	14 (16.3)
History of prior abdominal surgery	40 (57.0)
History of prior ERCP/PTCD	56 (53.5)
PLA under laid-in biliary stent	33 (38.4)
Initial symptoms^a^	
Fever	51 (59.3)
Right upper quadrant pain	41 (47.7)
Chills	14 (16.3)
Jaundice	10 (11.6)
Unspecific abdominal pain	6 (7.0)
Other	30 (34.9)
None	3 (3.5)
Number of abscess	
One	57 (66.3)
Two	12 (14.0)
Multiple	17 (19.8)
Size of abscess (diameter)	
< 5 cm	25 (29.1)
5–10 cm	33 (38.4)
> 10 cm	11 (12.8)
Not documented	17 (19.8)

*Abbreviations*: *ERCP* endoscopic retrograde cholangiopancreatography, *PTCD* percutaneous transhepatic cholangiography and drainage

^a^Patients fit to plural categories were counted in each category

As expected from a hospital with a major hepatobiliary surgery and liver transplant center, most of the PLA were of secondary nature originating in bile duct stenosis/obstruction (31.4%), anastomosis leakage and biliary infection (both 15.1%) as well as a superinfected liver metastasis (9.3%). In 14 patients (16.3%) the cause of liver abscess remained cryptogenic.

The most frequent comorbidities included malignancies in 34.9%, diabetes in 24.4%, the presence of liver cirrhosis in 16.3%, and prior liver transplantation in 16.3% of cases. Of note, 74.4% and 17.4% of patients received proton-pump inhibitors and immunosuppressive therapy prior to PLA formation, respectively.

### Microbiological investigations

**Fig. 2:**
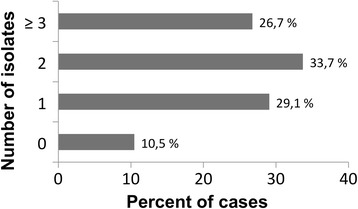
Number of bacterial isolates recovered per case in patients with pyogenic liver abscess

Microbiological cultures (blood and/or abscess cavity cultures) were set up in all 86 patients and were positive in 77 out of 86 (89.5%). Mycotic coinfections were documented in 21 cases (24.4%), mostly caused by *Candida albicans* (76.2% of all mycotic coinfections). The number of recovered bacterial/mycotic species per patient is represented in Fig. 2. Overall, 135 bacterial isolates were identified; a detailed overview is displayed in Table 3. In short, both gram-negative (48.9%) and gram-positive (46.7%) aerobic bacteria were frequently cultured, whereas anaerobic bacteria were identified relatively rarely (4.4%). Of note, the most common isolated bacterial species detected were *Enterococcus* spp. (28.9%, in total: *E. faecium* 26; *E. faecalis* 13). Patients’ characteristics with cultures positive for *Enterococcus* spp. and bivariate analysis are displayed in Table 4. In multivariate analysis, there was a trend to Enterococcus infections in patients taking proton-pump inhibitors (*P* = 0.057, OR 3.73, 95% CI 0.96–14.41). ERCP/PTCD in the last three months (*P* = 0.010, OR 4.23, 95% CI 1.41–12.77) and history of prior abdominal surgery (*P* = 0.036, OR 3.36, 95% CI 1.08–10.44) were independently associated with *Enterococcus* spp. infection in patients with PLA.

**Table 3:** Bacterial isolates from abscess cavity cultures

Bacterial isolates^a^	Number of isolates
Gram-positive aerobes	
*Staphylococcus aureus*	2
Coagulase neg. Staphylococci	13
Viridans streptococci	7
Group A Streptococci	1
*Enterococcus* spp.	39
other gram-positive species	1
Gram-negative aerobes	
*Escherichia coli*	23
*Klebsiella* spp.	15
other Enterobacteriaceae	14
*Pseudomonas* spp.	6
*Stenotrophomonas* spp.	3
*Acinetobacter* spp.	1
other gram-negative species	4
Anaerobes	6
No bacterial growth	9

^a^There were 4 cases with negative abscess cavity culture and positive blood cultures only. In all cases the cultured isolates were directly associated with the underlying PLA

**Table 4:** Patients’ characteristics and baseline parameters with and without cultivated *Enterococcus* spp.

Characteristics	Patient with Enterococcus isolates (*n* = 37)	Patient without Enterococcus isolates (*n* = 41)^a^	P
Age, y	65 (56–72)	55 (50–70)	0.168
Duration of hospitalization, days	23 (14–37)	18 (9–30)	0.123
Biliary cause of PLA, n (%)	28 (75.7)	21 (35.0)	0.035
Malignancy, n (%)	16 (43.2)	14 (35.0)	0.491
Liver cirrhosis, n (%)	7 (18.9)	6 (15.0)	0.764
Diabetes mellitus, n (%)	11 (29.7)	9 (22.5)	0.604
Cholangitis, n (%)	20 (54.1)	17 (42.5)	0.365
Intake of immunosuppressants, n (%)	7 (18.9)	7 (17.5)	1.000
Intake of proton-pump inhibitors, n (%)	32 (86.5)	26 (65.0)	0.036
Previous known MDRO, n (%)	11 (29.7)	4 (10.0)	0.043
Previous admission <3 month, n (%)	35 (94.6)	30 (75.0)	0.026
Previous ICU admission <3 month, n (%)	11 (29.7)	6 (15.0)	0.170
Previous surgical/endoscopic Intervention			
Previous endoscopic Intervention, n (%)	29 (78.4)	22 (55.0)	0.053
Previous ERCP/PTCD, n (%)	29 (78.4)	17 (42.5)	0.002
ERCP/PTCD <3 month, n (%)	27 (73.0)	16 (40.0)	0.006
Laid-in biliary stent, n (%)	23 (62.2)	13 (32.5)	0.012
History of abdominal surgery, n (%)	28 (75.7)	17 (42.5)	0.005
Abdominal surgery <6 month, n (%)	16 (43.2)	12 (30.0)	0.246
Laboratory results			
C-reactive protein (mg/dl)	11.2 (6.2–14.7)	11.1 (8.9–18.0)	0.266
White blood count (/nl)	10.3 (7.0–17.9)	12.6 (9.2–16.2)	0.580
International normalized ratio	1.3 (1.1–1.5)	1.3 (1.2–1.4)	0.899
Creatinine (mg/dl)	0.81 (0.62–1.23)	0.79 (0.57–0.93)	0.303
Albumin (g/dl)	2.7 (2.1–3.2)	2.8 (2.4–3.0)	0.647
Aspartate aminotransferase (U/l)	68 (28–136)	44 (26–96)	0.365
Alanine aminotransferase (U/l)	40 (20–75)	37 (19–74)	0.947
γ-Glutamyltransferase (U/l)	202 (136–403)	203 (92–534)	0.828
Alkaline phosphatase (UI/l)	239 (143–401)	221 (138–432)	0.859
Bilirubin (mg/dl)	1.0 (0.6–5.2)	1.0 (0.6–3.6)	0.719

Data are presented as media (IQR) unless otherwise indicated

*Abbreviations*: *ERCP* endoscopic retrograde cholangiopancreatography, *PTCD* percutaneous transhepatic cholangiography and drainage

^a^Patients without bacterial isolates were excluded

Summaries of antimicrobial susceptibility and resistance profiles of enterococci, gram-positive and gram-negative aerobic bacteria are displayed in Table 5. Detailed resistance profiles of individual bacterial isolates are shown in Additional file 1: Table S1, susceptibility and resistance profiles of gram-positive aerobic bacteria excluding enterococci in Additional file 2: Table S3 and those of anaerobic bacteria in Additional file 3: Table S4. Overall, 25 MDROs were identified. Most interestingly, 35.9% of all *Enterococcus* spp. were classified as VRE (*E. faecium* 46.2%, *E faecalis* 7.7%) including cases of teicoplanin, daptomycin-, and linezolid- (intermediate) resistance (Table 5, Additional file 1: Table S1). Importantly, although the PLA patient populations were comparable over the study period, the relative VRE rate (VRE among all Enterococci) in patients with PLA increased over time (Additional file 4: Figure S1A and B), while in other patients without PLA it did not change significantly (Additional file 5: Figure S2A and B). In addition, 12 cases of MRGN were identified (16,7% of gram-negative bacteria). Of note, approximately 10% and 20% of tested gram-negative aerobe bacteria were resistant to carbapenems and to tigecycline, respectively. Resistance rates to fluoroquinolones, broad-spectrum penicillin/β-lactamase inhibitor combinations (BSP/βLI) and cephalosporins were relatively high (20–57%, Table 5). Results of fungal isolates analysis are displayed in Additional file 6: Table S2. Next, we analyzed both patient populations with and without MDRO causing PLA. Results of bivariate analyses are depicted in Table 6. Of note, in multivariate analysis prior known MDRO (*P* = 0.0002, OR 30.51, 95% CI 5.15–180.78) and the use of glycopeptide antibiotics prior to culture collection (*P* = 0.030, OR 6.46, 95% CI 1.20–34.87) were independently associated with MDRO causing PLA in our patients.

**Table 5:** Summary of susceptibility profiles of aerobic bacteria

All gram-positive aerobes including *Enterococc*us spp.				
Antibiotics	Susceptible (%)	Intermediate (%)	Resistant (%)	Total:
Ampicillin	21 (44.7)	0 (0)	26 (55.3)	47
Amox/Clav.	21 (44.7)	0 (0)	26 (55.3)	47
Pip/Taz.	21 (47.7)	0 (0)	23 (52.3)	44
Cefuroxime	12 (19.0)	0 (0)	51 (81.0)^a^	63
Cefotaxime	10 (20.4)	0 (0)	39 (79.6)^a^	49
Gentamycin	27 (51.9)	0 (0)	25 (48.1)	52
Tigecycline	42 (100)	0 (0)	0 (0)	42
Levofloxacin	21 (35.0)	2 (3.3)	37 (61.7)	60
Vancomycin	43 (76.8)	0 (0)	13 (23.2)	56
Imipenem	13 (34.2)	0 (0)	25 (65.8)	38
Linezolid	44 (97.8)	1 (2.2)	0 (0)	45
*Enterococcus* spp. only				
Antibiotics	Susceptible (%)	Intermediate (%)	Resistant (%)	Total:
Ampicillin	12 (31.6)	0 (0)	26 (68.4)	38
Erythromycin	9 (25.0)	3 (8.3)	24 (66.7)	36
Imipenem	13 (34.2)	0 (0)	25 (65.8)	38
Tigecycline	32 (100)	0 (0)	0 (0)	32
Vancomycin	21 (60.0)	0 (0)	14 (40.0)	35
Teicoplanin	35 (92.1)	1 (2.6)	2 (5.3)	38
Levofloxacin	10 (26.3)	1 (2.6)	27 (71.1)	38
Linezolid	33 (97.1)	1 (2.9)	0 (0)	34
Daptomycin	7 (87.5)	0 (0)	1 (12.5)	8
All gram-negative aerobes				
Antibiotics	Susceptible (%)	Intermediate (%)	Resistant (%)	Total:
Ampicillin	8 (15.1)	1 (1.9)	44 (83.0)	53
Amox/Clav	17 (33.3)	5 (9.8)	29 (56.9)	51
Pip/Taz	30 (52.6)	6 (10.5)	21 (36.8)	57
Cefuroxime	17 (33.3)	8 (15.7)	26 (51.0)	51
Cefotaxime	30 (60.0)	0 (0)	20 (40.0)	50
Ceftazidim	10 (66.7) (52.2)	2 (13.3)	3 (20.0)	15
Imipenem	53 (84.1)	3 (4.8)	7 11.1)	63
Meropenem	55 (90.2)	1 (1.6)	5 (8.2)	61
Gentamicin	52 (83.9)	0 (0)	10 (16.1)	62
Tigecycline	35 (71.4)	4 (8.2)	10 (20.4)	49
TMP/SMX	42 (73.7)	0 (0)	15 (26.3)	57
Levofloxacin	42 (68.9)	2 (3.3)	17 (27.9)	61
Ciprofloxacin	41 (68.3)	1 (1.7)	18 (30.0)	60

*Amox/Clav* amoxicillin/clavulanic acid, *Pip/Taz* piperacillin/tazobactam, *TMP/SMX* trimethoprim/sulfamethoxazole

^a^ *Enterococcus* spp. with an intrinsic resistance

**Table 6:** Patients’ characteristics and baseline parameters with and without cultivated multidrug-resistant organisms

Characteristics	Patient with MDRO isolates (*n* = 21)	Patient without MDRO isolates (*n* = 52)^a^	P
Age, y	61 (52–71)	63 (52–73)	0.812
Duration of hospitalization, days	20 (13–37)	19 (10–32)	0.425
Biliary cause of PLA, n (%)	16 (76.2)	32 (61.5)	0.284
Malignancy, n (%)	7 (33.3)	22 (42.3)	0.600
Liver cirrhosis, n (%)	4 (19.0)	8 (15.4)	0.734
Diabetes mellitus, n (%)	4 (19.0)	16 (30.8)	0.392
Cholangitis, n (%)	14 (66.7)	22 (42.3)	0.074
Intake of immunosuppressants, n (%)	6 (28.6)	14 (26.9)	1.000
Intake of proton-pump inhibitors, n (%)	17 (81.0)	39 (75.0)	0.762
Previous known MDRO, n (%)	12 (57.1)	3 (5.8)	<0.0001
Previous admission <3 month, n (%)	20 (95.2)	44 (84.6)	0.432
Previous ICU admission <3 month, n (%)	7 (33.3)	10 (19.2)	0.229
Previous surgical/endoscopic Intervention			
Previous endoscopic Intervention, n (%)	17 (81.0)	33 (63.5)	0.174
Previous ERCP/PTCD, n (%)	17 (81.0)	28 (53.8)	0.036
ERCP/PTCD <3 month, n (%)	17 (81.0)	25 (48.1)	0.017
Laid-in biliary stent, n (%)	14 (66.7)	22 (42.3)	0.074
History of abdominal surgery, n (%)	15 (71.4)	29 (55.8)	0.293
Abdominal surgery <6 month, n (%)	9 (42.9)	18 (34.6)	0.023
Prior antibiotic therapy^b^			
Any antibiotic therapy, n (%)	15 (78.9)	26 (55.3)	0.096
Glycopeptide based, n (%)	7 (36.8)	4 (8,5)	0.010
Carbapenem based, n (%)	10 (52.6)	12 (25.5)	0.046
Piperacillin/Tazobactam, n (%)	4 (21.1)	4 (8.5)	0.213
Laboratory results			
C-reactive protein (mg/dl)	9.78 (6.9–15.2)	11.28 (8.6–20.8)	0.453
White blood count (/nl)	10.4 (7.5–15.6)	11.6 (7.7–16.4)	0.943
International normalized ratio	1.2 (1.1–1.4)	1.3 (1.2–1.4)	0.165
Creatinine (mg/dl)	0.91 (0.62–1.20)	0.79 (0.58–0.95)	0.352
Albumin (g/dl)	2.6 (2.2–2.9)	2.8 (2.2–3.1)	0.500
Aspartate aminotransferase (U/l)	69 (35–106)	42 (24–113)	0.328
Alanine aminotransferase (U/l)	40 (25–64)	34 (18–84)	0.933
γ-Glutamyltransferase (U/l)	281 (136–441)	199 (93–458)	0.371
Alkaline phosphatase (UI/l)	282 (196–401)	226 (133–425)	0.247
Bilirubin (mg/dl)	1.0 (0.5–4.9)	1.0 (0.6–3.4)	0.981

Data are presented as media (IQR) unless otherwise indicated

*Abbreviations*: *ERCP* endoscopic retrograde cholangiopancreatography, *PTCD* percutaneous transhepatic cholangiography and drainage

^a^Patients without resistance profile of bacterial isolates or without bacterial isolates were excluded

^b^Defined as ≥72 h antibiotic therapy before given cultures were attempted, overall *n* = 66

### Treatment of PLA

As initial empiric antibiotic therapy, 48.8% of included patients received a carbapenem-based regimen, 22.4% of patients BSP/βLI, and 17.6% of patients a third generation cephalosporin. In 32.6% and 8.1% of patients, a glycopeptide antibiotic or tigecycline was added to initial empirical treatment, respectively. 12 patients (14.0%) were treated with an additional antimycotic agent upon diagnosis. Of note, 35.7% of patients had received an inappropriate initial empirical antibiotic treatment, as evidenced by subsequent microbiological culture results.

Only 7 patients (8.1%) were solitary medically managed. Almost all patients received a percutaneous drainage of the abscess cavity (43.0% CT-guided, 47.7% US-guided). Additional interventional ERCP/PTCD was performed in 25.6% of cases. Surgery was necessary in 9.3% of cases.

### Complications & outcome

The median duration of hospitalization was 20 days. Recurrent hepatic abscesses after discharge were reported in 18 patients (20.9%).

Twenty-four patients (27.9%) required intensive care therapy. Results of uni- and multivariate analysis are depicted in Table 7. Of note, only diabetes mellitus (*P* = 0.048, OR 3.72, 95% CI 1.01–13.70) and mycotic coinfection (*P* = 0.012, OR 5.54, 95% CI 1.46–21.06) as well a carbapenem based initial empirical antibiotic therapy (*P* = 0.037, OR 3.73, 95% CI 1.09–12.89) independently predicted an ICU stay during hospitalization.

**Table 7:** Predictors of intensive care unit stay during hospitalization

	Univariate analysis		Multivariate analysis	
Variables	OR (95% CI)	*P* value	OR (95% CI)	*P* value
Age	1.01 (0.97–1.04)	0.69		
Male gender	0.88 (0.32–2.43)	0.81		
Predisposing disease				
Malignancy	1.06 (0.39–2.88)	0.91		
Liver cirrhosis	3.38 (1.02–11.22)	0.047	3.45 (0.79–14.99)	0.1
Diabetes mellitus	3.50 (1.21–10.14)	0.021	3.72 (1.01–13.70)	0.048
Medication				
Proton-pump inhibitor use	1.39 (0.44–4.39)	0.57		
Immunosuppression	4.11 (1.27–13.37)	0.019		
Blood values				
C-reactive Protein	1.02 (0.97–1.07)	0.37		
WBC	1.03 (0.97–1.09)	0.29		
Bilirubin	1.06 (0.95–1.18)	0.29		
Creatinine	1.82 (0.87–3.82)	0.11		
INR	6.74 (0.67–67.24)	0.10		
Cholangitis	1.64 (0.62–4.36)	0.32		
MDRO	1.08 (0.36–3.29)	0.89		
Mycotic coinfection	3.92 (1.33–11.55)	0.013	5.54 (1.46–21.06)	0.012
Initial empirical antibiotic treatment				
Carbapenem based	4.37 (1.49–12.81)	0.007	3.73 (1.09–12.89)	0.037
Glycopeptide based	3.35 (1.21–9.25)	0.019		
Tigecycline based	2.14 (0.43–10.58)	0.35		
Metronidazole based	0.42 (0.11–1.64)	0.21		

Fourteen patients died during their hospital stay, resulting in an overall mortality rate of 16.3%. In univariate and multivariate analysis (Table 8), increased bilirubin levels (multivariate *P* = 0.015, OR 0.85, 95% CI 0.75–0.97) and presence of malignancy (multivariate *P* = 0.041, OR 0.19, 95% CI 0.04–0.94) were significantly associated with mortality of PLA. Of note, neither the MDRO status nor the correct initial empiric therapy was related to ICU stay or survival.

**Table 8:** Predictors of survival in patients suffering from pyogenic liver abscess

	Univariate analysis		Multivariate analysis	
Variables	OR (95% CI)	*P* value	OR (95% CI)	*P* value
Age	0.99 (0.95–1.04)	0.73		
Male gender	0.86 (0.22–3.38)	0.83		
Predisposing disease				
Malignancy	0.22 (0.05–0.93)	0.04	0.19 (0.04–0.94)	0.041
Liver cirrhosis	0.77 (0.14–4.18)	0.77		
Diabetes mellitus	1.38 (0.27–7.19)	0.70		
Medication				
Proton-pump inhibitor use	0.68 (0.13–3.52)	0.64		
Immunosuppression	0.85 (0.16–4.57)	0.85		
Blood values				
C-reactive Protein	1.10 (0.98–1.23)	0.10		
WBC	0.98 (0.91–1.06)	0.56		
Bilirubin	0.85 (0.75–0.98)	0.02	0.85 (0.75–0.97)	0.015
Creatinine	0.63 (0.26–1.53)	0.30		
INR	0.44 (0.10–1.99)	0.29		
Cholangitis	0.19 (0.04–0.98)	0.046		
MDRO	0.75 (0.17–3.26)	0.70		
Mycotic coinfection	0.41 (0.10–1.67)	0.22		
Initial empirical antibiotic treatment				
Carbapenem based	0.60 (0.15–2.33)	0.46		
Glycopeptide based	1.12 (0.26–4.79)	0.88		
Tigecycline based	0.79 (0.08–7.56)	0.84		
Metronidazole based	2.89 (0.33–25.03)	0.33		

## Discussion

In this study, we provide detailed characteristics of PLAs with a special focus on bacterial pathogens causing PLA in a large German tertiary reference center. We observed a so far underreported role of *Enterococcus* spp*.* and other MDRO in the pathogenesis of secondary PLA, and present – to the best of our knowledge – a unique analysis of current bacterial susceptibility profiles from a large tertiary reference center in a Western country, which may be utilized to guide empirical antibiotic treatment of secondary PLA.

Of note, marked differences between PLA with patients in Western and Asia countries have been uncovered [16]. In our study, as in earlier reports from Western countries [1–3, 16], malignant and non-malignant biliary disease was the most identifiable direct cause of PLA. In Asian countries, the causes of PLAs often remain cryptogenic and the most commonly isolated bacterium is *Klebsiella pneumoniae* [5, 17–19]. Similarly to other Western reports [1–4], we have observed a divergent bacterial spectrum, characterized by a plethora of gram-positive and gram-negative bacteria, which have to be taken into account for choosing optimal antibiotic regimens. Most interestingly, *Enterococcus* spp*.* were most frequently isolated (in 28.9% of cases) in our study. So far, *Enterococcus* was considered to play a negligible role in PLA in Asia [5, 17–19] as well as the Western World: In the latter, rates were accounted for less than 7.2% of patients [2, 4, 14], only one study documented rates of 13.9% [21]. Two Western reports summarized *Streptococcus* spp. and *Enterococcus* spp. as one group of isolates (no exact percentage of *Enterococcus* alone was indicated). By doing so higher rates were documented (22.6% and 29.5%) [3, 22].

*Enterococcus* spp*.* has been observed as one of the predominant bacterial pathogens in cholangitis, especially in the setting of therapeutic endoscopy or presence of biliary endoprosthesis [23–25]. In line with these findings, many PLA patients included in this study had already received a therapeutic endoscopy prior to PLA manifestation. Moreover, we observed that ERCP/PTCD three months prior to PLA occurrence was independently associated with positive cultures for *Enterococcus* spp.

Although enterococci belong to the physiological flora of the alimentary tract and are traditionally considered to be of low virulence, in more seriously ill patients enterococcal infections have been associated with higher risk of treatment failure and mortality and antimicrobial therapy is warranted [26]. Of note, most cultured enterococci in our study were resistant to commonly administered broad-spectrum antibiotics (e.g. piperacillin/tazobactam or imipenem). With an intrinsic resistance to cephalosporins and BSP as typical in case of *E. faecium*, therapy options are largely limited to glycopeptide antibiotics (vancomycin or teicoplanin), linezolid or daptomycin.

Over the past decades MDRO are increasingly being reported worldwide. VRE rates in Europe vary significantly. In a large survey of patients with healthcare associated infections, in approximately 10,1% of patients VRE was documented [27]. Comparable results were observed in a survey of 126 ICUs in the United States [28].

Of note, vancomycin resistance was detected in more than 1/3 of *Enterococcus* spp*.* in our PLA study (VRE-rate in *E. faecium* up to 46%) and we observed an increase of VRE over the study period. In addition, our study provides evidence for an increasing risk of resistant gram-negative bacteria in PLA. 16,7% of all gram-negative bacteria were classified as MRGN. Even more worrisome, approximately 11% of all gram-negative bacteria were resistant to carbapenems. In line with these observations, Lo et al. noted an increase of MDRGN from 1.64 in 2001 to 14.29% in 2011 in Singapore [14]. Finally, 50% of all anaerobes isolated in our study were resistant to metronidazole. Yet, the relevance of this finding remains unclear because these isolates (*Propionibacterium acnes*) may be contaminants rather than causative bacteria.

Interestingly, MDRO cultivated in PLA patients were strongly associated with prior history of MDRO and more often found after prior antibiotic therapy, especially after the use of glycopeptide antibiotics. Moreover, neither the MDRO status nor the correct initial empiric therapy was associated with ICU stay or survival. In view of this data, MDRO may reflect severity of illness rather than being a predictor of mortality.

However, in a setting with a high prevalence of *Enterococcus* and VRE infection, as described, it appears necessary to treat critically ill patients with PLA with a combination of a carbapenem and an antibiotic targeting *Enterococcus* such as teicoplanin until microbiological test results are available. To avoid further spreads of resistance, rigorous de-escalation strategies appear to be warranted and stable patients may be empirically treated with a third-generation cephalosporin in combination with an agent against anaerobes. Careful screening for *Enterococcus* spp. and MRDO infections would be required in that scenario.

Limitations of our study remain in its retrospective design, based on a single diagnosis code with a relatively small study population with 86 patients in total. Furthermore, it was performed in a single major hepatobiliary surgery and liver transplant center. Thus, the spectrum of disease may reflect more the specific patient population and hence, not all observations and conclusions may be generalizable. However, it provides valuable information in a setting of growing numbers in biliary/abdominal surgery and endoscopic interventions.

## Conclusion

Our study demonstrates a so far underreported role of *Enterococcus* spp. in secondary. A worrisome number of VRE and other MRGN such as *Escherichia coli* and *Klebsiella pneumoniae* have been observed. Patients on PPI, or with prior ERCP/PTCD, or history of abdominal surgery appear to be at higher risk for VRE, and those with a prior history of MDRO infection at considerably higher risk for MDRO as a cause of PLA. Thus, thorough microbiological diagnostics is pivotal to tailor individual treatment regimens in order to prevent further selection of bacterial resistance in PLA, a diagnosis in which long durations of antibiotic therapy are often required.

## Additional files

Additional file 1: Table S1. Overview of bacterial isolates and their susceptibility profile. (XLSX 68 kb)

Additional file 2: Table S3. Susceptibility profiles of gram-positive aerobes, excluding *Enterococcus* supp. (DOCX 50 kb)

Additional file 3: Table S4. Susceptibility profiles of anaerobic bacteria. (DOCX 39 kb)

Additional file 4: Figure S1. MDRO rate (MDRO among all PLA patient) and relative VRE rate (VRE among all Enterococci) in patients with PLA. A marked increase of VRE causing PLA was observed. Data is represented per year (A) and with retrospect to defined study periods (B). (TIFF 12962 kb)

Additional file 5: Figure S2. Relative VRE rate (VRE among all Enterococci) of all reported Enterococcus infections among patients without PLA. Local hospital surveillance data in our gastroenterology/hepatology wards revealed no increase in the relative VRE rate among all non-PLA patients. Results are represented per year (A), and with retrospect to defined study periods (B). (TIFF 11180 kb)

Additional file 6: Table S2. Overview of fungal isolates and their susceptibility profile. (XLSX 47 kb)

## Abbreviations

βLI: β-lactamase inhibitor
BSP: Broad-spectrum penicillin
CI: Confidence interval
CT: Computed tomography
ERCP: Endoscopic retrograde cholangiography
ICU: Intensive care unit
MDROs: Multidrug-resistant organisms
MRGN: Multidrug-resistant gram-negative bacteria
MRI: Magnetic resonance imaging
MRSA: Methicillin-resistant *Staphylococcus aureus*
OR: Odds ratio
PLA: Pyogenic liver abscess
PTCD: Percutaneous transhepatic cholangiography and drainage
US: Ultrasound
VRE: Vancomycin-resistant enterococci
