# Supplementary material for: The role of Enterococcus spp. and multidrug-resistant bacteria causing pyogenic liver abscesses
Source: BMC Infect Dis. 2017 Jun 26;17:450. doi: 10.1186/s12879-017-2543-1 (PMC5485679; doi:10.1186/s12879-017-2543-1)
Supplement: Supplementary file 2 — Susceptibility profiles of gram-positive aerobes, excluding Enterococcus supp. (DOCX 50 kb) [file 12879_2017_2543_MOESM2_ESM.docx]

| **Antibiotics** | **Susceptible (%)** | **Intermediate (%)** | **Resistant (%)** | **Total:** |
| --- | --- | --- | --- | --- |
| Penicillin | 10 (45.5) | 0 (0) | 12 (54.5) | 22 |
| Oxacillin | 2 (15.4) | 0 (0) | 11 (84.6) | 13 |
| Ampicillin | 9 (100) | 0 (0) | 0 (0) | 9 |
| Amox/Clav | 9 (100) | 0 (0) | 0 (0) | 9 |
| Pip/Taz | 9 (100) | 0 (0) | 0 (0) | 9 |
| Cefuroxime | 12 (52.2) | 0 (0) | 11 (47.8) | 23 |
| Cefotaxime | 9 (100) | 0 (0) | 0 (0) | 9 |
| Gentamycin | 10 (43.5) | 0 (0) | 13 (56.5) | 23 |
| Tigecycline | 10 (100) | 0 (0) | 0 (0) | 10 |
| Levofloxacin | 11 50.0) | 1 (4.5) | 10 (45.5) | 22 |
| Vancomycin | 21 (100) | 0 (0) | 0 (0) | 21 |

**Table S3.** Susceptibility profiles of gram-positive aerobes, excluding *Enterococcus supp.*

Amox/Clav: amoxicillin/clavulanic acid; Pip/Taz: piperacillin/tazobactam
