# Supplementary material for: The role of Enterococcus spp. and multidrug-resistant bacteria causing pyogenic liver abscesses
Source: BMC Infect Dis. 2017 Jun 26;17:450. doi: 10.1186/s12879-017-2543-1 (PMC5485679; doi:10.1186/s12879-017-2543-1)
Supplement: Supplementary file 3 — Susceptibility profiles of anaerobic bacteria. (DOCX 39 kb) [file 12879_2017_2543_MOESM3_ESM.docx]

**Table S4:** Susceptibility profiles of anaerobic bacteria.

| **Anaerobes** | **Susceptible (%)** | **Intermediate (%)** | **Resistant (%)** | **Total** |
| --- | --- | --- | --- | --- |
| Pip/Taz | 4 (100) | 0 (0.0) | 0 (0.0) | 4 |
| Imipenem | 4 (100) | 0 (0.0) | 0 (0.0) | 4 |
| Metronidazole | 2 (50.0) | 0 (0.0) | 2 (50.0) | 4 |
| Clindamycin | 2 (100) | 0 (0.0) | 0 (0.0) | 2 |

Pip/Taz: piperacillin/tazobactam
