# Supplementary figures and images for: The role of Enterococcus spp. and multidrug-resistant bacteria causing pyogenic liver abscesses
Source: BMC Infect Dis. 2017 Jun 26;17:450. doi: 10.1186/s12879-017-2543-1 (PMC5485679; doi:10.1186/s12879-017-2543-1)

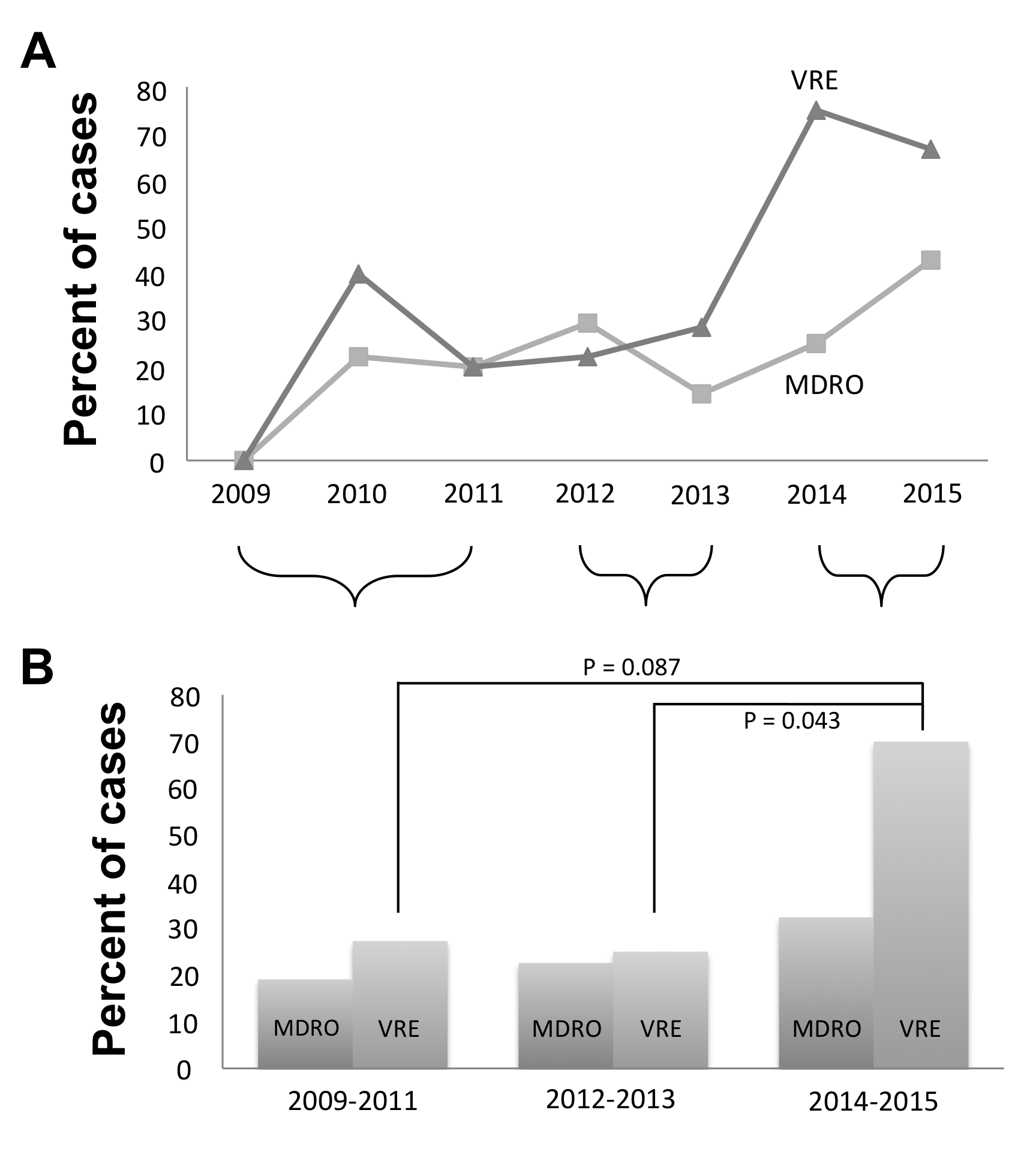

Supplement: Supplementary file 4 — MDRO rate (MDRO among all PLA patient) and relative VRE rate (VRE among all Enterococci) in patients with PLA. A marked increase of VRE causing PLA was observed. Data is represented per year (A) and with retrospect to defined study periods (B). (TIFF 12962 kb) [file 12879_2017_2543_MOESM4_ESM.tif]

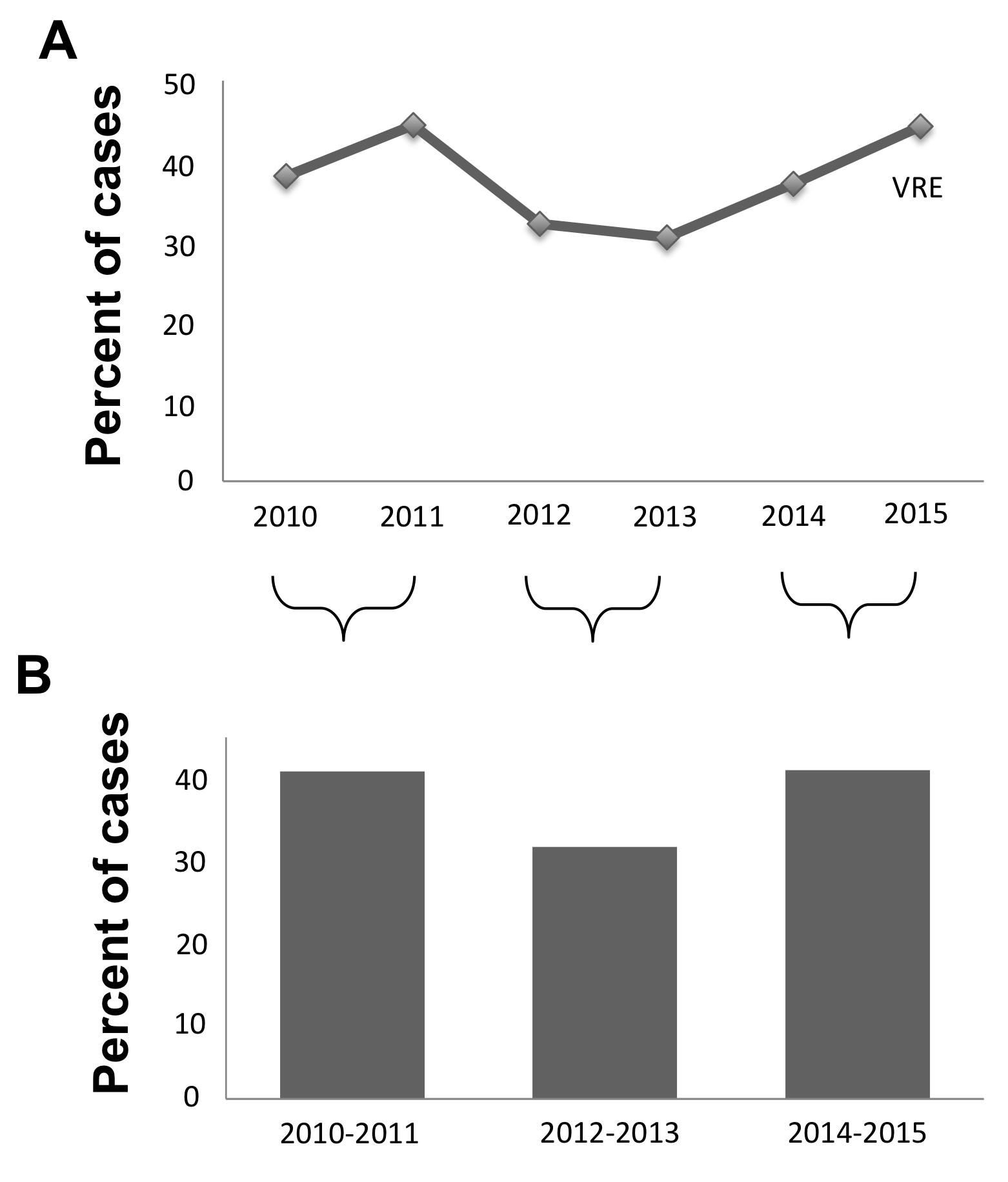

Supplement: Supplementary file 5 — Relative VRE rate (VRE among all Enterococci) of all reported Enterococcus infections among patients without PLA. Local hospital surveillance data in our gastroenterology/hepatology wards revealed no increase in the relative VRE rate among all non-PLA patients. Results are represented per year (A), and with retrospect to defined study periods (B). (TIFF 11180 kb) [file 12879_2017_2543_MOESM5_ESM.tif]
